# A partial supernumerary umbilical vein: a case report

**DOI:** 10.1186/s13256-019-2094-8

**Published:** 2019-05-18

**Authors:** Mariko Kurakazu, Masamitsu Kurakazu, Masaharu Murata, Tatsuki Miyamoto, Yoko Takahashi, Makoto Hamasaki, Eiji Ohta, Fusanori Yotsumoto, Shingo Miyamoto

**Affiliations:** 1 Department of Obstetrics and Gynecology, Faculty of Medicine, Fukuoka University, Fukuoka, Japan; 2 Center for Maternal, Fetal and Neonatal Medicine, Fukuoka University Hospital, Fukuoka, Japan; 3 Department of Pathology, Faculty of Medicine, Fukuoka University, Fukuoka, Japan

**Keywords:** Case report, Congenital anomaly, Four-vessel umbilical cord, Prenatal diagnosis, Single umbilical artery, Supernumerary umbilical vein

## Abstract

**Background:**

Abnormalities in the number of vessels can be found for both the umbilical artery and vein. We sometimes encounter cases of a decreased number of umbilical cord vessels, such as a single umbilical artery. In contrast, there may be an increase from three to four vessels within the umbilical cord. A supernumerary umbilical vein is particularly very rare, and it is generally found in combination with congenital anomalies. We report a case of a partial supernumerary umbilical vein.

**Case presentation:**

The previous pregnancy of a 37-year-old healthy Japanese woman (gravida 2, para 1) had been uncomplicated, and the resulting child was alive and well. Prenatal examination at 36 weeks of gestation revealed the coexistence of a four-vessel part and a normal three-vessel part of the umbilical cord. A healthy female neonate weighing 2726 g was born at 38 weeks of gestation. The umbilical cord measured 40 cm in length; the four-vessel part continued to a distance of 18 cm from the surface of the infant’s body, and the remaining umbilical cord comprised three vessels. On histological examination, the fetal side of the umbilical cord had two arteries and two veins, and the placental side had two arteries and one vein. Isolated supernumerary umbilical veins tend to be overlooked. We consider that it is important to evaluate the number of umbilical cord vessels in the second trimester using ultrasound combined with color Doppler in at least three sites: the insertion sites on both the fetal abdomen and placenta, and the free loop of the umbilical cord.

**Conclusions:**

Prenatal diagnosis of isolated supernumerary umbilical cord vessels tends to be overlooked. However, supernumerary vessels of the umbilical can be associated with fetal congenital anomalies. The number of vessels within the umbilical cord must be examined because the detection of such abnormalities may lead to the prenatal diagnosis of other congenital anomalies.

## Background

Abnormalities in the number of vessels can be found for both the umbilical artery and vein. We sometimes encounter cases of a decreased number of umbilical cord vessels, such as a single umbilical artery (SUA). A SUA is associated with fetal growth restriction and cardiac and renal anomalies [1]. In contrast, there may be an increase from three to four vessels within the umbilical cord. Meyer *et al*. [2] reported that it is more common to encounter an accessory fourth vessel of the umbilical cord than a SUA. Although a supernumerary umbilical vein is reportedly associated with a very high incidence of congenital anomalies [3], there are several reports of very rare isolated cases of a supernumerary umbilical cord (two arteries and two veins) without any fetal abnormality [4]. We report a case of a partial four-vessel (two arteries and two veins) umbilical cord.

## Case presentation

**Fig. 1:**
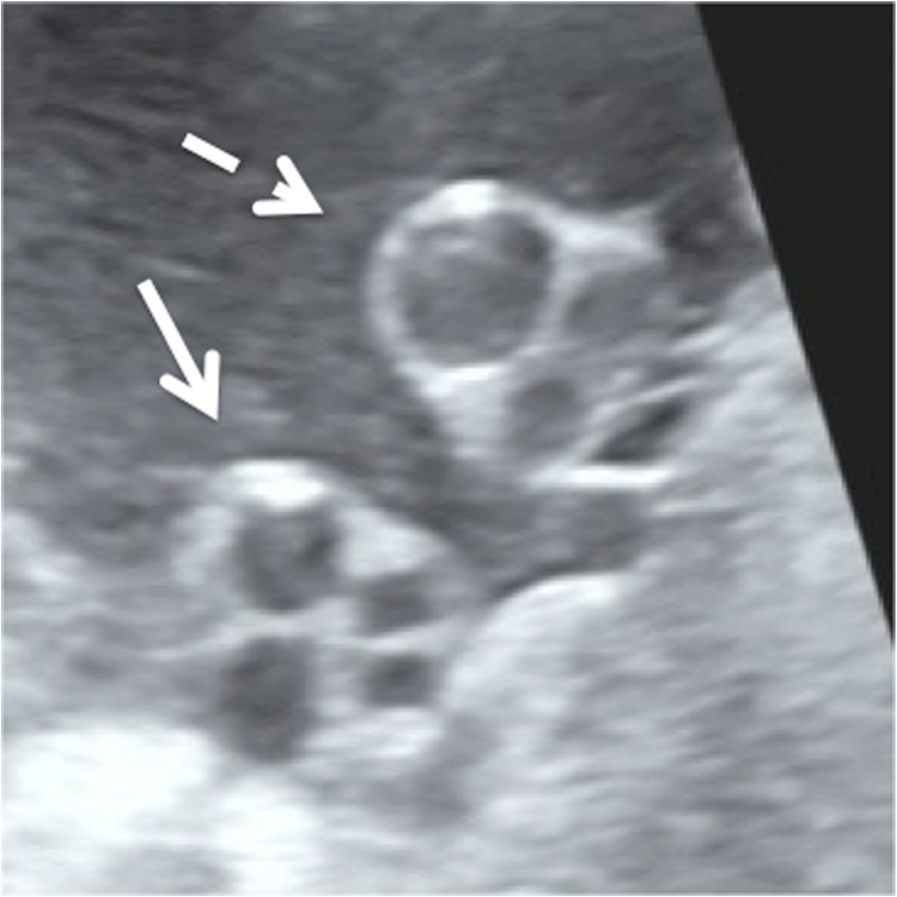
Prenatal ultrasonography shows the four-vessel (*arrow*) and three-vessel (*broken arrow*) parts of the free loop of the umbilical cord

**Fig. 2:**
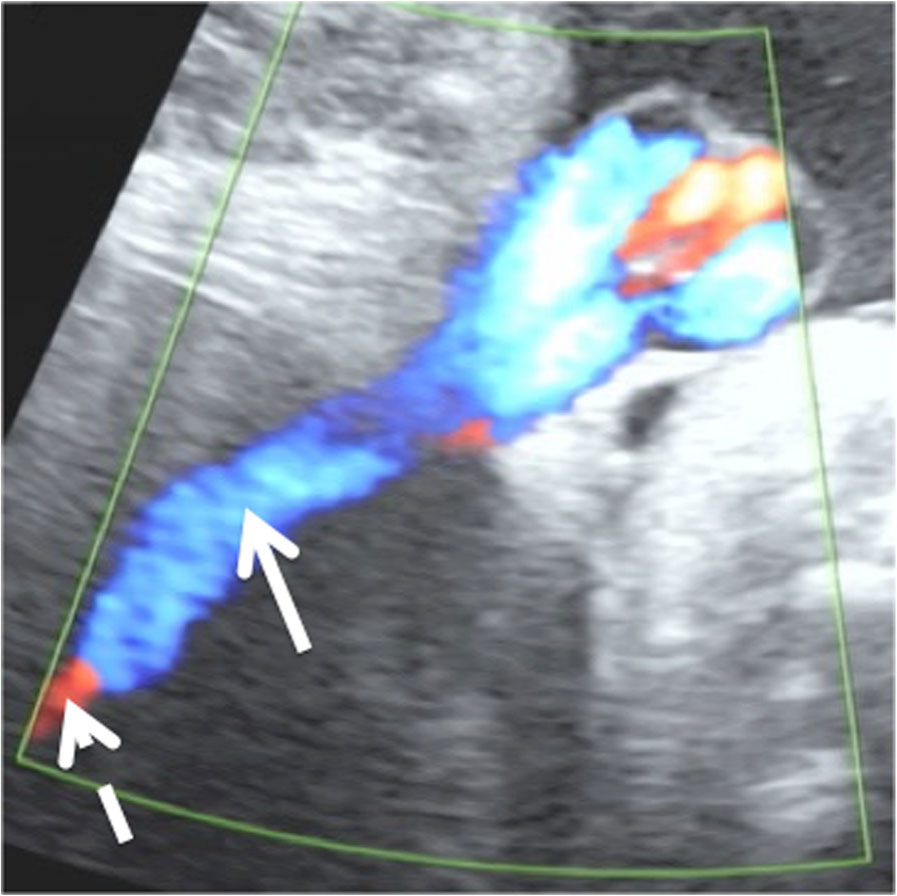
Transabdominal ultrasonography with color Doppler. The umbilical vein (*arrow*) and ductus venosus (*broken arrow*) are shown. The intra-abdominal umbilical vein is a single vessel connected to the ductus venosus

The previous pregnancy of a 37-year-old healthy Japanese woman (gravida 2, para 1) had been uncomplicated, and the resulting child was alive and well. She had no history of periconceptional tobacco smoking, alcohol intake, radiation exposure, or intake of other supplements and drugs. She had no clinical problems during the early period of the current pregnancy; however, she did not undergo an ultrasound examination during the first trimester. At 36 weeks of gestation of the current pregnancy, a routine ultrasound examination revealed an abnormal number of umbilical cord vessels. A fetal ultrasound examination was performed using a GE Voluson™ E10 ultrasound machine (General Electric Healthcare, Tokyo, Japan) with a 3.5-MHz convex-array transducer. The examination revealed the coexistence of a four-vessel part and a three-vessel part within the free loop of the umbilical cord (Fig. 1). The fetal insertion site of the umbilical cord comprised four vessels (two arteries and two veins), whereas the placental insertion site comprised three vessels (two arteries and one vein). The blood flow was demonstrated in both veins by two sonographic specialists in maternal fetal medicine, and the flow was similar in each vessel. However, we were unable to prenatally detect the exact point at which the umbilical cord changed from a four-vessel to a three-vessel cord. The intra-abdominal umbilical vein was a single vessel that was connected to the ductus venosus and returned to the right atrium (Fig. 2). No other sonographic congenital abnormalities were detected on fetal ultrasound screening performed in accordance with the recommendations of the International Society of Ultrasound in Obstetrics and Gynecology [5].

At 38 weeks of gestation, a healthy female neonate weighing 2726 g was delivered by spontaneous vaginal delivery. The infant’s Apgar scores were 9 and 10 at 1 minute and 5 minutes, respectively. The neonatal physical examination at birth was normal.

**Fig. 3:**
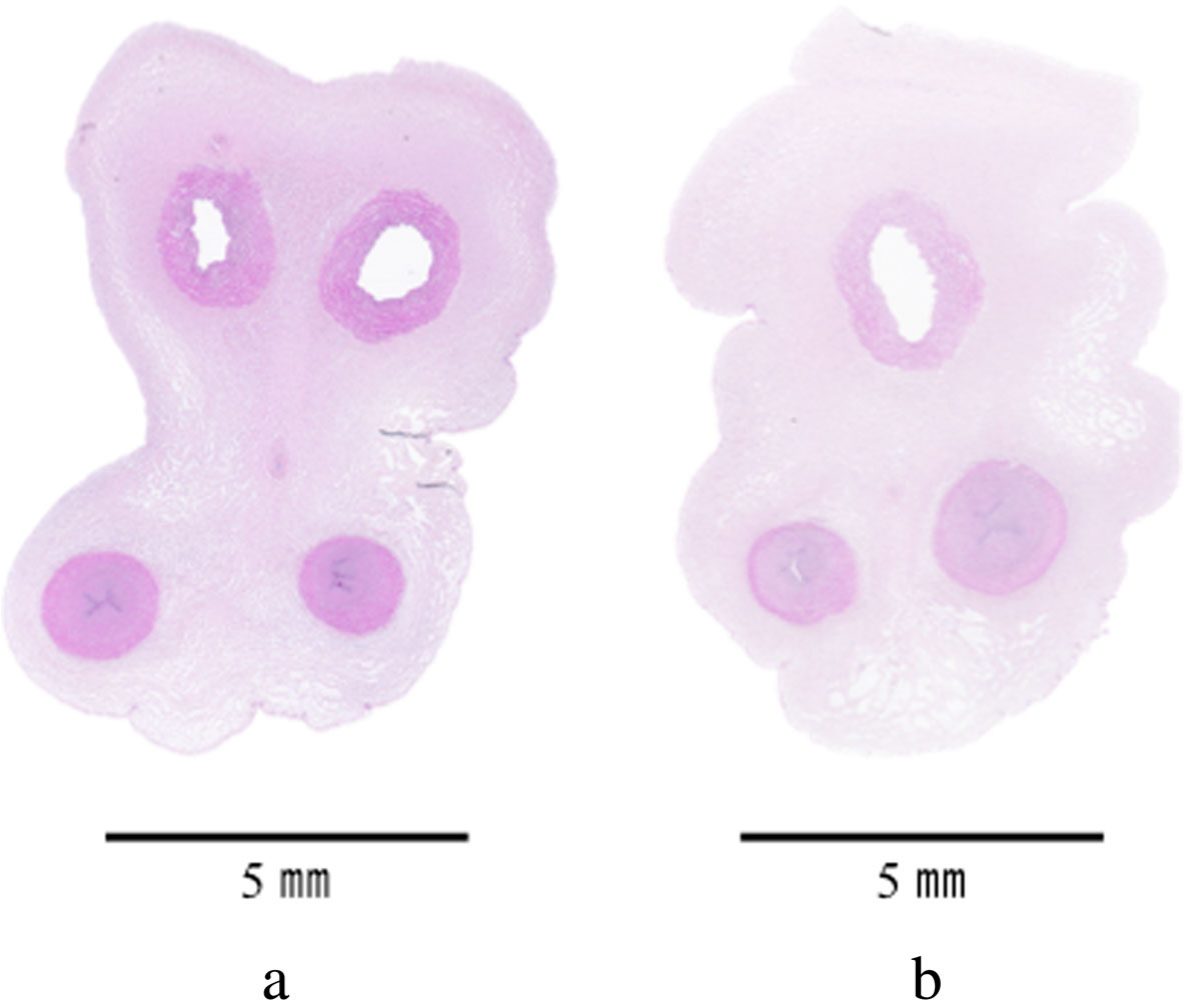
A hematoxylin and eosin-stained section shows the umbilical cord. **a** The upper two-vessel part is the vein, while the other two-vessel part is the artery. **b** Normal vessels (the upper two arteries and the other single vein)

Ultrasonographic examination of the infant at the age of 54 days revealed normal anatomy and no congenital anomalies. The umbilical cord measured 40 cm in length; the four-vessel part continued to a distance of 18 cm from the surface of the infant’s body, and the remaining umbilical cord comprised three vessels. On histological examination, the fetal side of the umbilical cord had two arteries and two veins, and the placental side had two arteries and one vein. Microscopic examination of cross-sectional sections of the umbilical cord obtained at 1 cm intervals and stained with both hematoxylin-eosin (Fig. 3a and b) and Elastica van Gieson revealed that the umbilical cord had a four-vessel part comprising two arteries and two veins, and that the two umbilical veins then fused, resulting in a three-vessel part comprising two arteries and one vein. The placenta had a trimmed weight of 430 g, and was both macroscopically and microscopically unremarkable.

## Discussion

In the middle of the fourth week of gestation, the sinus venosus receives blood from the right and left sinus horns [6, 7]. Each sinus horn receives blood from three important veins: the vitelline or omphalomesenteric vein, the umbilical vein, and the common cardinal vein [6, 7]. During the fourth and fifth weeks of development, the sinus shifts to the right and the left sinus horn is rapidly lost [6, 7]. A complex pattern of vessel growth occurs, and the left vitelline and umbilical veins atrophy and disappear [6, 7]. Failure of this process results in a persistent right umbilical vein (PRUV) [8].

A PRUV usually replaces the normal left umbilical vein or is supernumerary [9]. Schimmel and Eidelman [10] and Avnet *et al*. [11] reported that a supernumerary umbilical vein results in a four-vessel umbilical cord. In contrast, Canavan and Hill [12] reported that 24 of 313 infants with PRUV had a four-vessel umbilical cord. Murdoch [13] reported an anomalous cord containing two umbilical veins and a segment of double cord. In the present case, we found a normal left umbilical vein with a four-vessel umbilical cord. Several variations of supernumerary umbilical veins have been described; however, the pathogenesis of such variations remains unknown.

The present case of an isolated four-vessel umbilical cord was diagnosed in the third trimester. Lei *et al*. [14] reported that the presence of a four-vessel umbilical cord was diagnosed prenatally as the sole issue in seven of 16 cases; in the remaining nine cases, a four-vessel umbilical cord was diagnosed in combination with fetal growth restriction, oligohydramnios, and a variety of anomalies such as bilateral cleft lip and palate, esophageal atresia, atrioventricular septal defect, PRUV, hypertrophic cardiomyopathy, anterior chest wall defect, and heterotaxy syndrome. It is considered difficult to diagnose an abnormal number of umbilical vessels prenatally, even if other congenital abnormalities coexist. While a SUA can be easily detected by the presence of only one umbilical artery lateral to the fetal bladder, the presence of supernumerary umbilical veins tends to be overlooked, as it is often difficult to check the number of umbilical veins throughout the length of the umbilical cord. We consider that it is important to evaluate the number of umbilical cord vessels in the second trimester using ultrasound combined with color Doppler in at least three sites: the insertion sites on both the fetal abdomen and placenta, and at more than one point in the free loop of the umbilical cord.

## Conclusions

Prenatal diagnosis of isolated supernumerary umbilical cord vessels tends to be overlooked. However, supernumerary vessels of the umbilical can be associated with fetal congenital anomalies. The number of umbilical cord vessels must be examined because abnormalities may suggest the presence of congenital anomalies.

## Abbreviations

PRUV: Persistent right umbilical vein
SUA: Single umbilical artery
